# Sacubitril/Valsartanstive Heart Failure: Cardiogenic Shock

**DOI:** 10.1155/2018/8231576

**Published:** 2018-05-15

**Authors:** H. A. Rawal, A. G. Kocheril

**Affiliations:** ^1^Internal Medicine, University of Illinois at Urbana-Champaign, 611 West Park St., Urbana, IL 61801, USA; ^2^Department of Cardiovascular Diseases and Clinical Electrophysiology, Christie Clinic, 1400 West Park St., Urbana, IL 61801, USA

## Abstract

Sacubitril/valsartan is a combination drug described as a new class of dual-acting angiotensin receptor-neprilysin inhibitor (ARNi) for heart failure. We present a case of a patient with NYHA class IV systolic heart failure who was refractory to all other classes of heart failure medications and was started on this new medication. On sacubitril/valsartan, he developed cardiogenic shock. This led us to reevaluate the use and risks of this medication in the class IV heart failure population.

## 1. Background

In the American Heart Association (AHA)/American College of Cardiology (ACC) guidelines [1], congestive heart failure (CHF) is defined as “a complex clinical syndrome that can result from any structural or functional cardiac disorder that impairs the ability of the ventricle to fill or eject blood” [1]. In the US, the prevalence of HF exceeds 5.8 million, and the incidence is >550,000/year [2]. ACE inhibitors (ACEIs) and beta-blockers have been associated with decreased mortality in clinical trials. There is consensus that both of these medications are complementary and can be started at the same time as soon as the diagnosis of HF is made.

Sacubitril/valsartan (initially referred to as LCZ696) is an orally acting supramolecular sodium salt complex of the neprilysin inhibitor prodrug sacubitril and the angiotensin receptor blocker (ARB) valsartan, which was recently approved for the treatment of chronic heart failure (NYHA classes II–IV) with reduced ejection fraction (HFrEF) [3]. This drug has been studied in patients with heart failure with both preserved ejection fraction (HFpEF) and reduced ejection fraction (HFrEF). It has shown to reduce mortality in patients with HFrEF; however, its mortality benefits in HFpEF still need to be studied as per the PARAMOUNT trial.

## 2. Case

**Figure 1:**
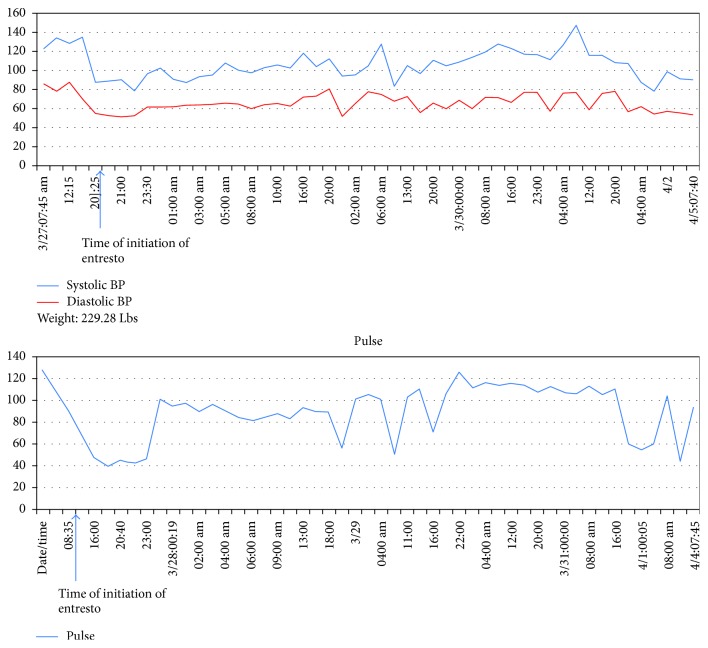


A 76-year-old male with a history of systolic CHF, NYHA class IV, presented with worsening shortness of breath over 4 weeks and fleeting chest pains. He mentioned having orthopnea and cough with blood-tinged sputum. On examination, he was tachycardiac, and heart rate was irregular, but other vitals were stable; he did not have jugular venous distention but had basilar crackles. He also had a history of coronary artery disease and an implantable cardioverter-defibrillator (ICD) for secondary prevention. EKG showed atrial fibrillation (AF) with a rapid ventricular rate and a right bundle branch block. Labs were unremarkable except an elevated B-type natriuretic peptide (BNP). A recent echocardiogram demonstrated an ejection fraction of 22% with global left ventricular hypokinesis. His St. Jude Medical Fortify Assura VR single-lead ICD was interrogated and was functioning normally. Along with diuresis, he was started on IV diltiazem drip for his uncontrolled AF. He had been intolerant to guideline-directed heart failure therapy in the past. Prior to his admission, treatment with lisinopril resulted in severe cough, losartan caused symptomatic hypotension, and carvedilol caused shortness of breath. Since his BP was 137/85, a decision was made to start the patient on low-dose sacubitril/valsartan without any other medications given the intolerance to routine HF medications. Within 2 doses and 24 hours of starting the medication, he had an episode of severe hypotension requiring pressor support for cardiogenic shock (Figure 1). Sacubitril/valsartan was discontinued since it was the only vasoactive medication the patient was receiving at that time for his heart failure, and subsequently, amiodarone was used for rhythm control in AF. While off that medication, the patient was treated for his cardiogenic shock, which included dobutamine. The blood pressure recovered transiently followed by a persistent drop despite maximal efforts. The family decided to withdraw care given his refractory cardiogenic shock. The time between starting sacubitril/valsartan and the time of death of the patient was 11 days.

## 3. Discussion

In the large, randomized, double-blind PARADIGM-HF trial, sacubitril/valsartan has demonstrated substantial reductions in all-cause mortality due to heart failure without altering its safety profile when compared to the standard treatment for heart failure [4]. On comparing sacubitril/valsartan with enalapril, there was a greater incidence of symptomatic hypotension but less angioedema [3]. ACE inhibitors are typically available in various doses, which can be titrated, whereas sacubitril/valsartan is available only in two doses. As per the ACC/AHA recommendations, sacubitril/valsartan and ACE/ARB should not be given within 36 hours of each other to reduce the risk of angioedema [3].

Besides the cardiac side effect profile, recently some light has been shed on the noncardiac side effects of this medication with long-term use. These include amyloid deposition in the eye and the brain, which theoretically can lead to visual and cognitive impairment [5, 6]. From previous studies, these side effects were considered only theoretical since it would likely take 10 to 20 years of use with this medication before the actual signs of disease were seen and the lifespan of most patients being on the drug was usually less than the time it may take for the long term neurocognitive side effects to develop [5, 6]. Hence, these side effects were given little importance. However, a recent letter in response to the statements suggested that eligible patients aged 55 years from the PARADIGM-HF trial had a projected life expectancy of 11.6 years while receiving enalapril and 12.9 years with sacubitril/valsartan, a difference of 1.3 years. Similar results were reported in eligible patients aged 65 years (10 years for enalapril versus 11.4 years for sacubitril/valsartan) [4–6]. These survivals clearly do indicate that the visual and cognitive side effects can be relevant. However, PARADIGM-HF trial did not have cognitive testing as a part of its follow-up in patients at 27 months. Hence, more research needs to be done so that we do not turn a blind eye to possible additional side effects of this medication [4–6].

## 4. Conclusion

We present a case of severe hypotension/cardiogenic shock that occurred after starting sacubitril/valsartan in a patient with NYHA grade IV heart failure. We feel the use of this medication should be limited to patients with NYHA class II and III systolic HF, which was most of the enrolled population in PARADIGM-HF. If any class IV patients were included, few patients who were hypotensive in the run-in period were not enrolled. As stated in the ACC/AHA guidelines, the incidence of severe hypotension and cardiogenic shock is likely to be higher in patients with NYHA class IV systolic heart failure, warranting at least added caution when using it in these patients. It is possible that visual and cognitive side effects will play an important role in patients who are on long-term use of this medication, thus likely necessitating regular testing.

## Additional Points

*Learning Points for Clinicians*. (1) Create more awareness about the use of this new heart failure medication in NYHA class IV patients. (2) Side effect profile including life-threatening complications like cardiogenic shock in advanced heart failure patients and non-life-threatening but concerning long-term effects like visual and cognitive impairment with prolonged use. (3) Necessity for further researches about potential long-term side effects of sacubitril/valsartan and its use for patients with diastolic heart failure.
